# Immunohistochemical Markers in Endometrial Cancer: Latest Updates

**DOI:** 10.3390/cancers15174202

**Published:** 2023-08-22

**Authors:** Valerio Mais, Michele Peiretti

**Affiliations:** 1 Department of Surgical Sciences, University of Cagliari Medical School, 09042 Cagliari, Italy; 2 Division of Gynecology and Obstetrics (AOU di Cagliari), Department of Surgical Sciences, University of Cagliari, 09042 Cagliari, Italy; mpeiretti@aoucagliari.it

Ten years ago, The Cancer Genome Atlas (TGCA) Research Network classified endometrial cancer into four molecular categories with prognostic significance, suggesting sensitivity to postsurgical treatments. This molecular classification is not used in clinical practice due to its high cost and the requirement of using fresh or frozen tissue [1]. Therefore, the PORTEC and ProMisE groups have improved the clinical applicability of the TGCA classification by identifying more affordable surrogates via the immunohistochemical assessment of mismatched repair (MMR) proteins and p53 in formalin-fixed paraffin-embedded (FFPE) tissue. Such surrogate classification defines four prognostic groups: POLE-mutated (POLEmut, which does not yet have an immunohistochemical surrogate), MMR-deficient (MMRd), p53-abnormal (p53abn), and non-specific molecular profile (NSMP) [1]. This Special Issue aims to provide an overview of the latest updates on the use of immunohistochemical markers to better characterize endometrial cancer in more homogeneous groups regarding prognosis and therapeutic strategies [1]. International leaders in the field of endometrial cancer immunohistochemistry and prognosis contributed a series of six articles (four original articles and two reviews) reporting recent advances in stratifying women with endometrial cancer into homogeneous prognostic groups who could benefit from specific individualized therapies [2,3,4,5,6,7].

Santoro et al. [2] conducted a valuable review by comparing all the described histological types of endometrial cancer with the PORTEC and ProMisE immunohistochemical surrogates of the prognostic molecular classification proposed by TCGA. Based on the authors’ review, the distribution of prognostic molecular groups in the different endometrial carcinoma histotypes showed some peculiar differences. Low-grade endometrioid endometrial carcinoma was associated with 5% of the cases in the p53abn group, 6% in the POLEmut group, 25% in the MMRd group, and 64% in the NSMP group. Conversely, the high-grade endometrioid endometrial carcinoma histotype was associated with 21% of the cases in the p53abn group, 12% in the POLEmut group, 39% in the MMRd group, and 28% in the NSMP group. This distribution was similar to that observed for undifferentiated/dedifferentiated endometrial carcinoma, which was associated with 19% of the cases in the p53abn group, 12% in the POLEmut group, 44% in the MMRd group, and 25% in the NSMP group. This distribution was not very different from that observed in neuroendocrine endometrial carcinoma, which was associated with 14% of the cases in the p53abn group, 7% in the POLEmut group, 43% in the MMRd group, and 36% in the NSMP group. In contrast, clear cell endometrial carcinoma was associated with 44% of the cases in the p53abn group and 42% in the NSMP group, but only 4% in the POLEmut group and 10% in the MMRd group. Carcinosarcoma was associated with 74% of the cases in the p53abn group and only 5%, 7%, and 14% in the POLEmut, MMRd, and NSMP groups, respectively. Finally, serous endometrial carcinoma was associated with practically 100% of the cases in the p53abn group. Moreover, within the NSMP group of endometrioid endometrial carcinomas, the absence of a specific molecular profile was associated with heterogeneous biological behavior, and the PORTEC group identified the expression of L1CAM and mutations in exon 3 of CTNNB1 as further independent prognostic factors [2].

Favier et al. [3] focused on the role of immunohistochemical markers in the characterization of the MMRd group of endometrial carcinomas. These authors conducted a systematic review to summarize the published literature on the immunohistochemical methodologies used to characterize this molecular group defined as MMRd, which was initially found in patients affected by a mutation known as Lynch syndrome (LS) [3]. In their review, Favier et al. [3] identified 10 papers that used immunohistochemistry to verify the loss of expression of two MMR system proteins (MLH1 and MSH2 proteins). They also identified 18 publications that used immunohistochemistry to verify the loss of expression of three MMR system proteins (MLH1, MSH2, and MSH6 proteins) and 96 papers that used immunohistochemistry to verify the loss of expression of four MMR system proteins (MLH1, MSH2, MSH6, and PMS2 proteins). Most of the identified publications analyzed FFPE tissue, and only a few publications analyzed frozen or fresh tissue [3]. Four publications described changes in PD-L1 expression to evaluate immune checkpoint pathways in MMRd endometrial carcinomas and reported an increase in PD-L1 expression [3]. Favier et al. [3] used immunohistochemistry to detect the expression of all four proteins, followed by MLH1 methylation analysis in MLH1-negative cases (to screen all women with endometrial cancer for LS), which remains the preferred procedure for diagnosing endometrial MMRd carcinomas. Additionally, recent studies have recognized MMRd endometrial cancers as having a better prognosis than non-MMRd cancers [3].

Assuming that women with both endometrial cancer and LS are typically younger than women with endometrial cancer but without LS, Pasanen et al. [4] tested whether limiting MLH1 methylation analysis to women below a certain age threshold could improve the cost-benefit ratio compared to the proposed age-independent universal screening. They tested tumor tissue samples from 842 women who underwent surgery at the Helsinki University Hospital over 5 years and from 142 women who were included in the Finnish national registry of LS patients. In terms of minimizing laboratory workload, limiting MLH1 methylation analysis to women younger than 60 years would have excluded just over 83% of women from the analysis. If the MLH1 methylation analysis had been limited to women under 65 years, this would have excluded almost 71% of women from the analysis. If the MLH1 methylation analysis had been limited to women under 70 years, this would have excluded just over 56% of women from the analysis [4]. Considering an age threshold of 60 years, the sensitivity of the method would have been 50% for the hospital-based cohort and 88.6% for the registry-based cohort. Considering an age threshold of 65 years, the sensitivity of the method would have been 97% for the hospital-based cohort and 100% for the registry-based cohort. A sensitivity equivalent to the latter would be obtained by considering a threshold age of 70 years. The authors concluded that in the Finnish population, limiting MLH1 methylation analysis to women older than 65 years could significantly reduce laboratory efforts while maintaining acceptable sensitivity [4].

Henry et al conducted research to verify whether the classification proposed by the PORTEC group was also applicable to the population of Aotearoa, a contemporary Maori-language name for New Zealand [5]. The authors then applied the PORTEC method to characterize the histological samples from 90 women affected by endometrial carcinoma; there were equal proportions of women of European origin (Euro, N = 30), women of Maori ethnicity (Maori, N = 30), and women from the islands of the Pacific Ocean (Pasifika, N = 30). Carcinoma histology was predominantly endometrioid (90–96%) in all three groups. Overall, of the 88 samples that were completed for analysis, 9% were POLEmut, 10% were P53abn, 17% were MMRd, and the vast majority (64%) were NSMP [5]. Furthermore, it should be emphasized that, despite the limited sample size in each ethnic group, CTNNB1 mutations were more frequent in the Maori and Pasifika groups than in the Euro group. Therefore, the authors suggested continuing the evaluation of this immunohistochemical marker to determine its potential inclusion in an Aotearoa-specific profiling panel, considering the high number of carcinomas in the NSMP category [5].

While exploring new immunohistochemical markers that may contribute to the prognostic characterization of endometrial cancer, Hojnik et al. [6] investigated the potential of aldo-keto reductase family 1 member B1 (AKR1B1) and aldo-keto reductase family 1 member B10 (AKR1B10) as tissue biomarkers in formalin-fixed, paraffin-embedded specimens from patients with either endometrioid endometrial cancer or serous endometrial cancer. This is because AKR1B1 plays a role in osmoregulation, prostaglandin synthesis, and the protein kinase C pathway, thus stimulating inflammation and cell proliferation. AKR1B10 also plays a role in cell proliferation and regulates fatty acid biosynthesis, which is involved in carcinogenesis. Hojnik et al. [6] evaluated the immunohistochemical (IHC) staining of AKR1B1 and AKR1B10 using validated antibodies in cancer tissue and adjacent nonneoplastic endometrial tissue. They also correlated AKR1B1 and AKR1B10 expression in cancer tissue with the clinicopathological data of patients with endometrioid endometrial cancer and demonstrated that cases with both AKR1B1 and AKR1B10 staining above the median had better overall and disease-free survival than cases with either or both AKR1B1 and AKR1B10 staining below the median [6]. The authors did not find significant correlations between AKR1B1 and AKR1B10 expression in cancer tissue and other clinical data. These results indicate that higher levels of both AKR1B1 and AKR1B10 staining in tissue from endometrioid endometrial cancer correlate with a better prognosis, suggesting a protective role of combined AKR1B1 and AKR1B10 actions and the possibility of using combined AKR1B1 and AKR1B10 staining as a new prognostic marker [6].

Eritja et al. [7] investigated the mechanisms by which PTEN mutations or deficiencies lead to the development of endometrial cancer. They used mouse endometrial organoids obtained from genetically modified mouse models and focused on the mechanisms by which the PTEN protein interacts with the SMAD2/3 proteins that regulate gene expression. They demonstrated that PTEN deficiency triggers a PI3K/AKT-dependent nuclear translocation of SMAD2/3, which functions independently of TGF-β receptor activation. Nuclear SMAD2/3 acts as a tumor suppressor and reduces the proliferation of endometrial cells induced by PTEN deficiency [7]. The authors also performed immunohistochemical analysis of formalin-fixed, paraffin-embedded samples from human endometrioid endometrial carcinoma tissues, including 19 grade 1, 23 grade 2, and 37 grade 3 carcinomas. Decreased PTEN expression was associated with an increase in nuclear staining for SMAD2/3 only in grade 3 endometrioid endometrial carcinoma tissues, suggesting that nuclear staining for SMAD2/3 could be used as an immunohistochemical marker of grade 3 endometrioid endometrial carcinomas with PTEN deficiency [7].

This series of valuable papers by researchers worldwide aims to provide useful information to clinicians and researchers. Indeed, clinicians must increasingly address the complexity of the realm of endometrial cancer to not only provide modern patient care but also design targeted therapeutic research protocols considering all diagnostic updates. Researchers should follow the insights offered by immunohistochemistry to identify new markers that will, hopefully soon, progressively reduce the percentage of carcinomas, which are currently defined as NSMP due to the absence of a specific molecular profile for this group.
